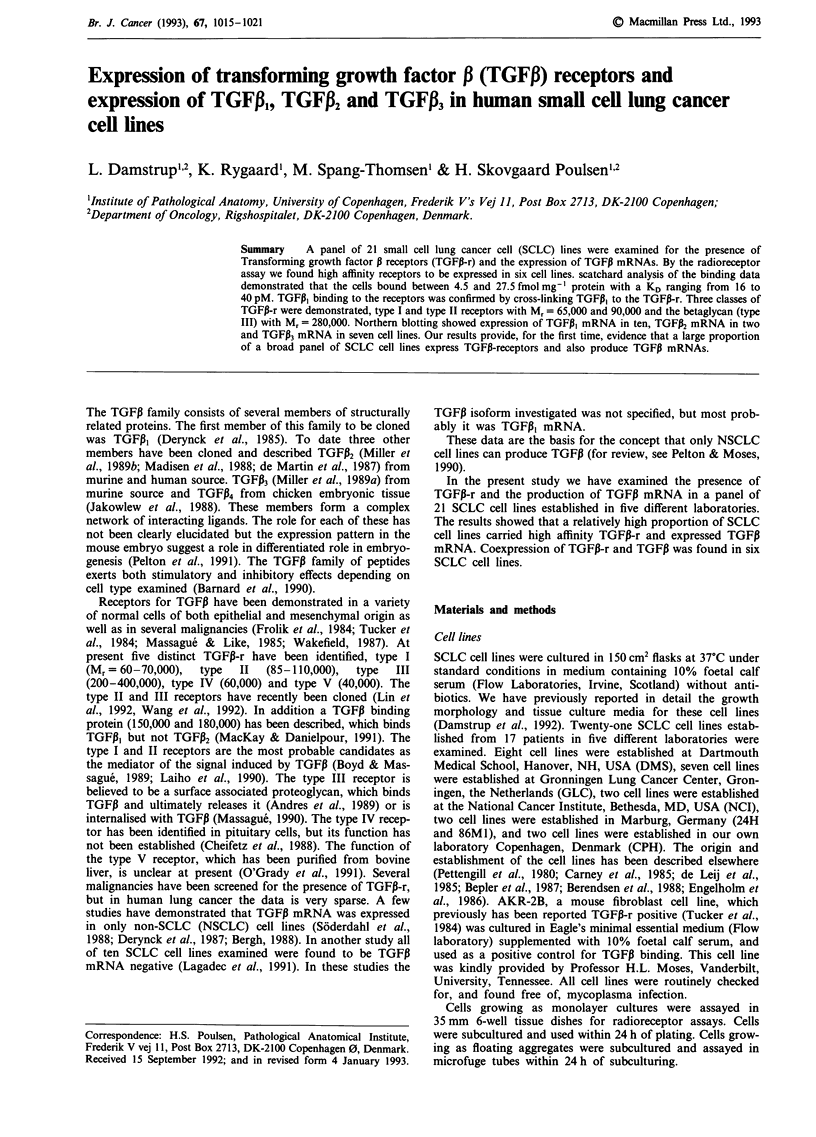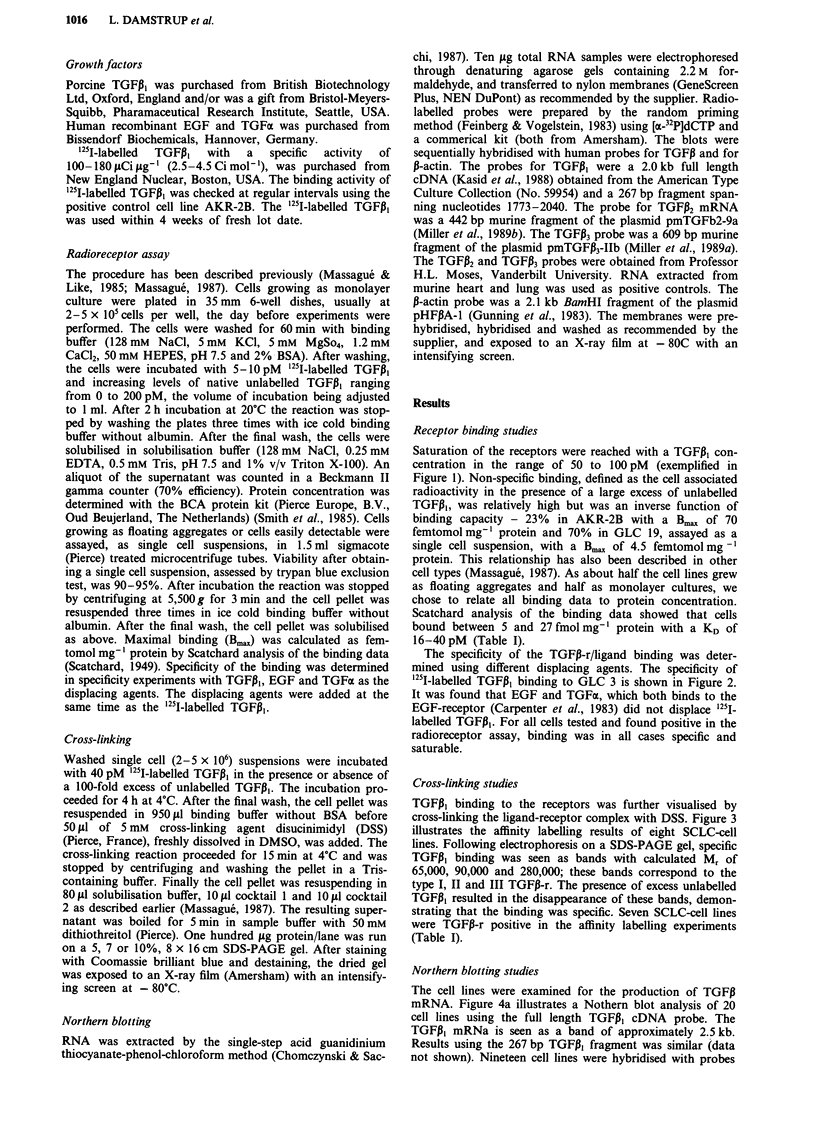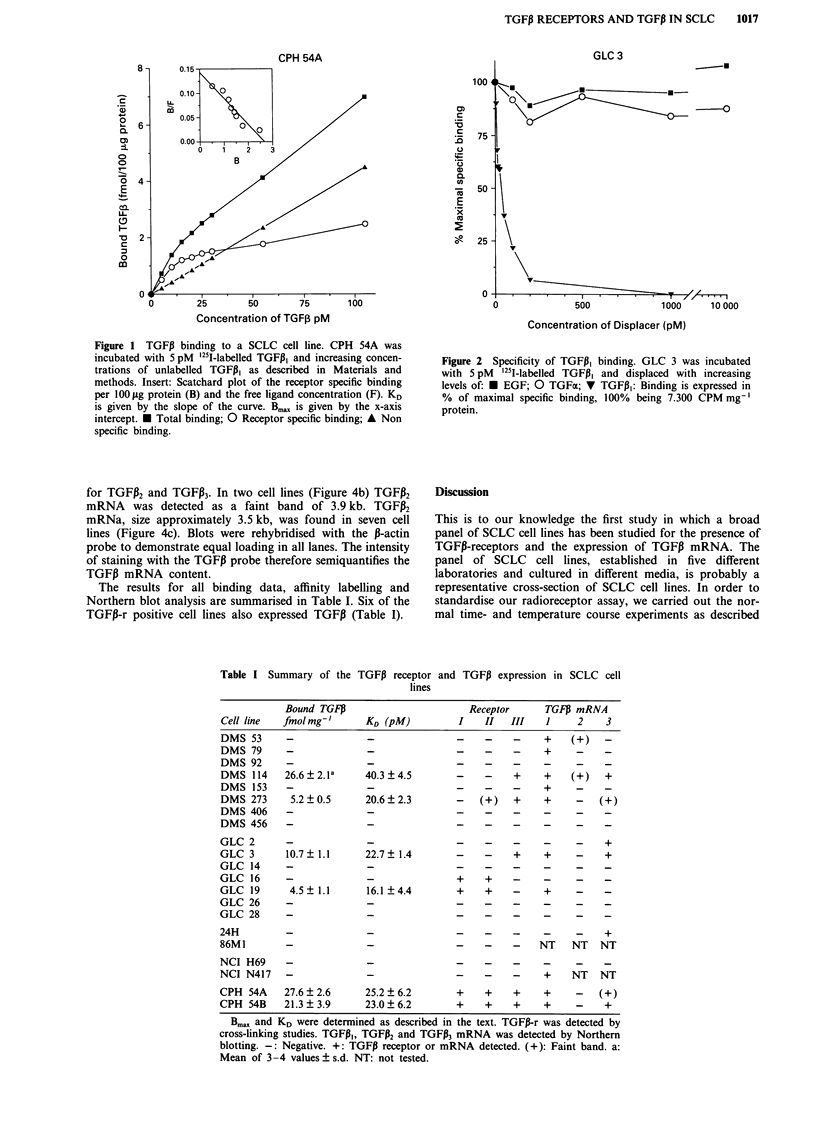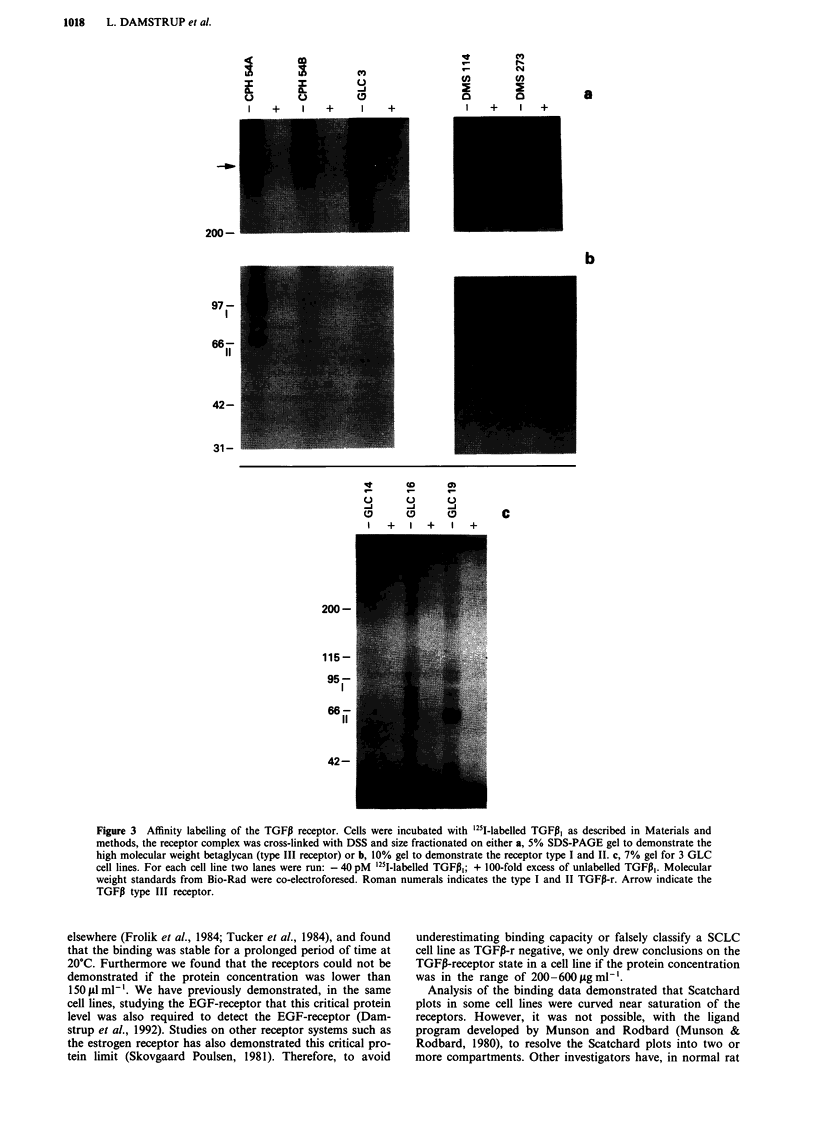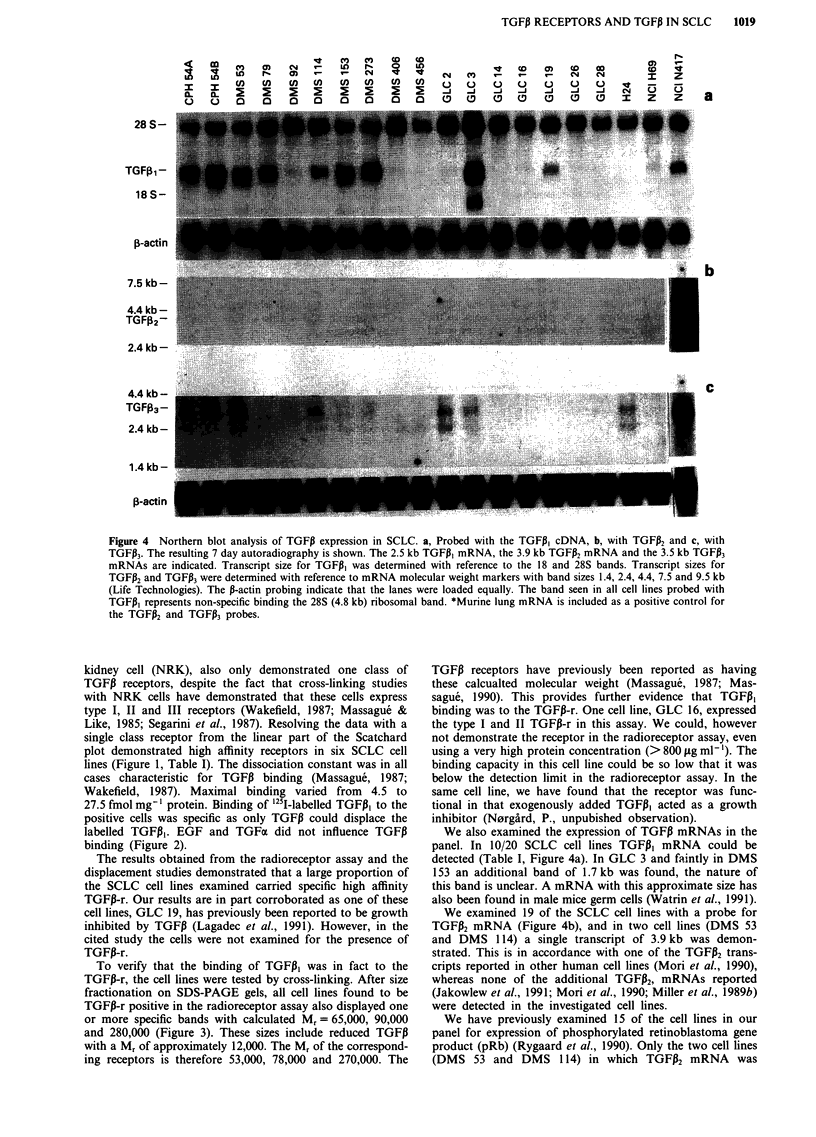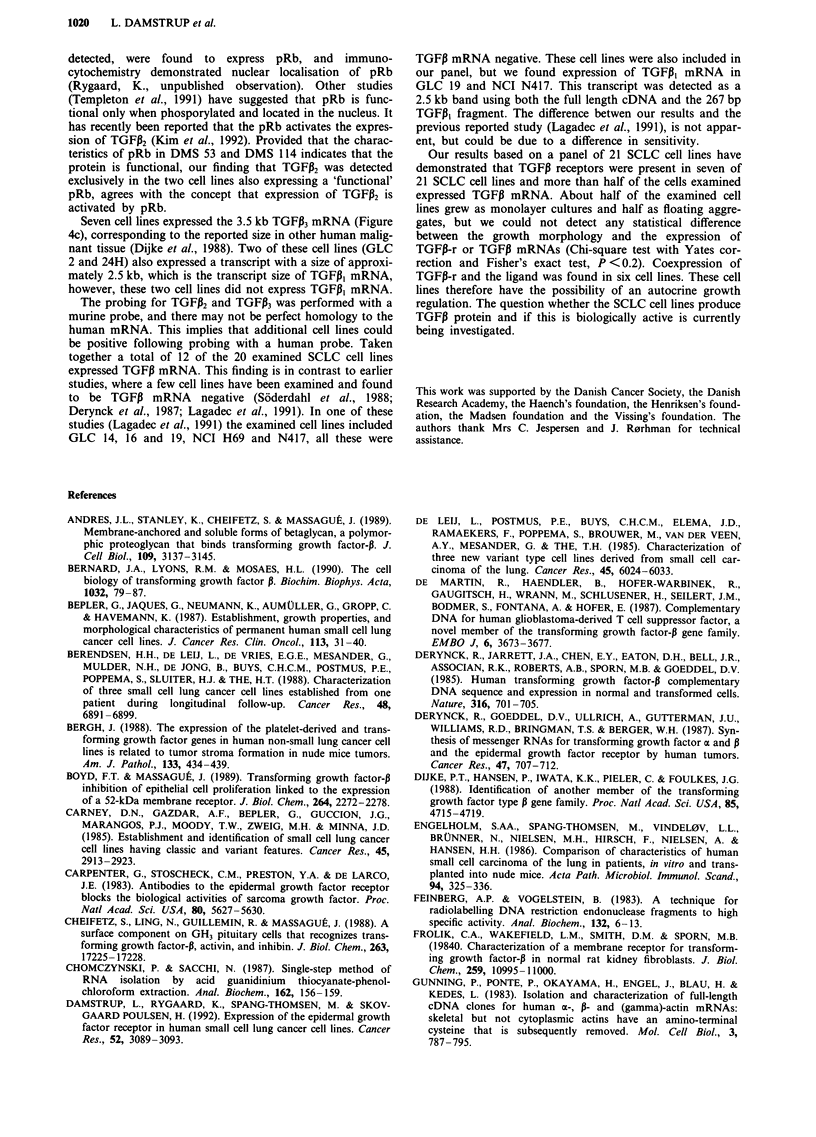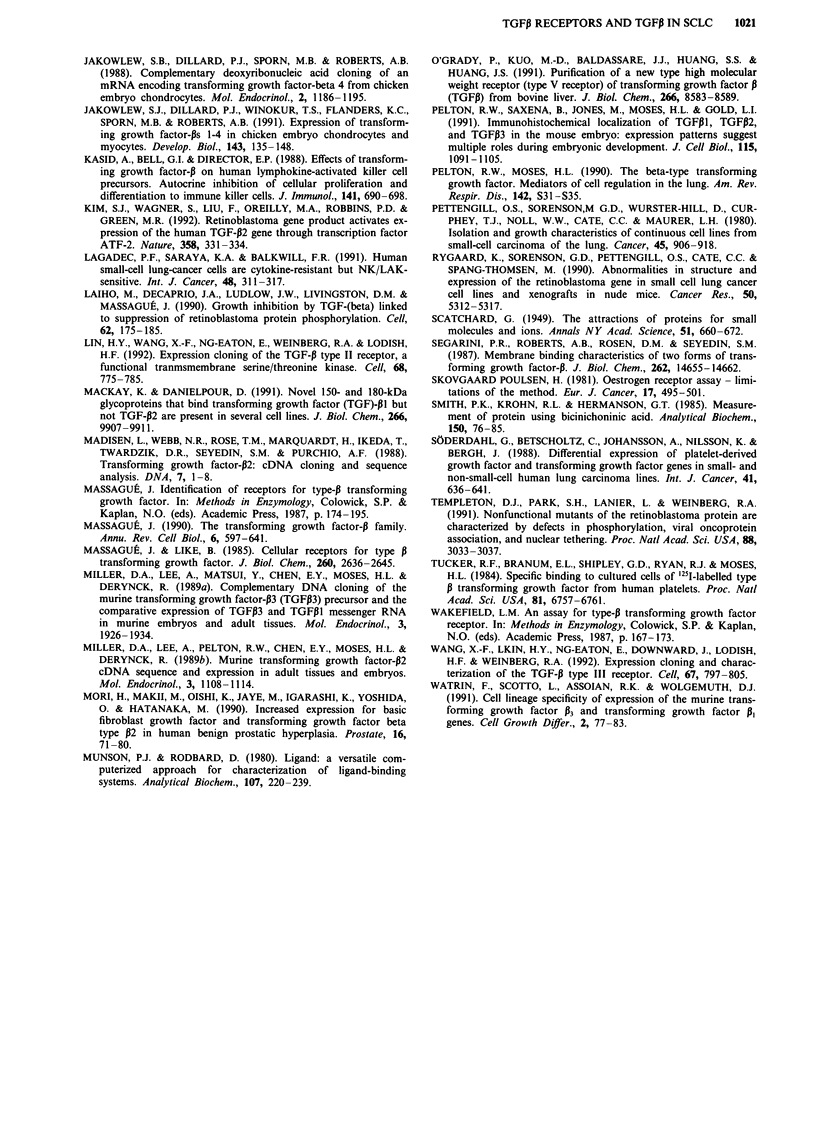# Expression of transforming growth factor beta (TGF beta) receptors and expression of TGF beta 1, TGF beta 2 and TGF beta 3 in human small cell lung cancer cell lines.

**DOI:** 10.1038/bjc.1993.186

**Published:** 1993-05

**Authors:** L. Damstrup, K. Rygaard, M. Spang-Thomsen, H. Skovgaard Poulsen

**Affiliations:** Institute of Pathological Anatomy, University of Copenhagen, Denmark.

## Abstract

**Images:**


*Br. J. Cancer* (1993), **67**, 1015–1021

© Macmillan Press Ltd., 1993

### Expression of transforming growth factor β (TGFβ) receptors and expression of TGFβ$_1$, TGFβ$_2$ and TGFβ$_3$ in human small cell lung cancer cell lines

L. Damstrup[1,2], K. Rygaard[1], M. Spang-Thomsen[1] & H. Skovgaard Poulsen[1,2]

[1]*Institute of Pathological Anatomy, University of Copenhagen, Frederik V's Vej 11, Post Box 2713, DK-2100 Copenhagen;* [2]*Department of Oncology, Rigshospitalet, DK-2100 Copenhagen, Denmark.*

**Summary** A panel of 21 small cell lung cancer cell (SCLC) lines were examined for the presence of Transforming growth factor β receptors (TGFβ-r) and the expression of TGFβ mRNAs. By the radioreceptor assay we found high affinity receptors to be expressed in six cell lines. scatchard analysis of the binding data demonstrated that the cells bound between 4.5 and 27.5 fmol mg$^{-1}$ protein with a $K_D$ ranging from 16 to 40 pM. TGFβ$_1$ binding to the receptors was confirmed by cross-linking TGFβ$_1$ to the TGFβ-r. Three classes of TGFβ-r were demonstrated, type I and type II receptors with $M_r = 65,000$ and 90,000 and the betaglycan (type III) with $M_r = 280,000$. Northern blotting showed expression of TGFβ$_1$ mRNA in ten, TGFβ$_2$ mRNA in two and TGFβ$_3$ mRNA in seven cell lines. Our results provide, for the first time, evidence that a large proportion of a broad panel of SCLC cell lines express TGFβ-receptors and also produce TGFβ mRNAs.

The TGFβ family consists of several members of structurally related proteins. The first member of this family to be cloned was TGFβ$_1$ (Derynck *et al.*, 1985). To date three other members have been cloned and described TGFβ$_2$ (Miller *et al.*, 1989*b*; Madisen *et al.*, 1988; de Martin *et al.*, 1987) from murine and human source. TGFβ$_3$ (Miller *et al.*, 1989*a*) from murine source and TGFβ$_4$ from chicken embryonic tissue (Jakowlew *et al.*, 1988). These members form a complex network of interacting ligands. The role for each of these has not been clearly elucidated but the expression pattern in the mouse embryo suggest a role in differentiated role in embryogenesis (Pelton *et al.*, 1991). The TGFβ family of peptides exerts both stimulatory and inhibitory effects depending on cell type examined (Barnard *et al.*, 1990).

Receptors for TGFβ have been demonstrated in a variety of normal cells of both epithelial and mesenchymal origin as well as in several malignancies (Frolik *et al.*, 1984; Tucker *et al.*, 1984; Massagué & Like, 1985; Wakefield, 1987). At present five distinct TGFβ-r have been identified, type I ($M_r = 60-70,000$), type II (85–110,000), type III (200–400,000), type IV (60,000) and type V (40,000). The type II and III receptors have recently been cloned (Lin *et al.*, 1992, Wang *et al.*, 1992). In addition a TGFβ binding protein (150,000 and 180,000) has been described, which binds TGFβ$_1$ but not TGFβ$_2$ (MacKay & Danielpour, 1991). The type I and II receptors are the most probable candidates as the mediator of the signal induced by TGFβ (Boyd & Massagué, 1989; Laiho *et al.*, 1990). The type III receptor is believed to be a surface associated proteoglycan, which binds TGFβ and ultimately releases it (Andres *et al.*, 1989) or is internalised with TGFβ (Massagué, 1990). The type IV receptor has been identified in pituitary cells, but its function has not been established (Cheifetz *et al.*, 1988). The function of the type V receptor, which has been purified from bovine liver, is unclear at present (O'Grady *et al.*, 1991). Several malignancies have been screened for the presence of TGFβ-r, but in human lung cancer the data is very sparse. A few studies have demonstrated that TGFβ mRNA was expressed in only non-SCLC (NSCLC) cell lines (Söderdahl *et al.*, 1988; Derynck *et al.*, 1987; Bergh, 1988). In another study all of ten SCLC cell lines examined were found to be TGFβ mRNA negative (Lagadec *et al.*, 1991). In these studies the TGFβ isoform investigated was not specified, but most probably it was TGFβ$_1$ mRNA.

These data are the basis for the concept that only NSCLC cell lines can produce TGFβ (for review, see Pelton & Moses, 1990).

In the present study we have examined the presence of TGFβ-r and the production of TGFβ mRNA in a panel of 21 SCLC cell lines established in five different laboratories. The results showed that a relatively high proportion of SCLC cell lines carried high affinity TGFβ-r and expressed TGFβ mRNA. Coexpression of TGFβ-r and TGFβ was found in six SCLC cell lines.

### Materials and methods

### *Cell lines*

SCLC cell lines were cultured in 150 cm$^2$ flasks at 37°C under standard conditions in medium containing 10% foetal calf serum (Flow Laboratories, Irvine, Scotland) without antibiotics. We have previously reported in detail the growth morphology and tissue culture media for these cell lines (Damstrup *et al.*, 1992). Twenty-one SCLC cell lines established from 17 patients in five different laboratories were examined. Eight cell lines were established at Dartmouth Medical School, Hanover, NH, USA (DMS), seven cell lines were established at Gronningen Lung Cancer Center, Groningen, the Netherlands (GLC), two cell lines were established at the National Cancer Institute, Bethesda, MD, USA (NCI), two cell lines were established in Marburg, Germany (24H and 86M1), and two cell lines were established in our own laboratory Copenhagen, Denmark (CPH). The origin and establishment of the cell lines has been described elsewhere (Pettengill *et al.*, 1980; Carney *et al.*, 1985; de Leij *et al.*, 1985; Bepler *et al.*, 1987; Berendsen *et al.*, 1988; Engelholm *et al.*, 1986). AKR-2B, a mouse fibroblast cell line, which previously has been reported TGFβ-r positive (Tucker *et al.*, 1984) was cultured in Eagle's minimal essential medium (Flow laboratory) supplemented with 10% foetal calf serum, and used as a positive control for TGFβ binding. This cell line was kindly provided by Professor H.L. Moses, Vanderbilt, University, Tennessee. All cell lines were routinely checked for, and found free of, mycoplasma infection.

Cells growing as monolayer cultures were assayed in 35 mm 6-well tissue dishes for radioreceptor assays. Cells were subcultured and used within 24 h of plating. Cells growing as floating aggregates were subcultured and assayed in microfuge tubes within 24 h of subculturing.

Correspondence: H.S. Poulsen, Pathological Anatomical Institute, Frederik V vej 11, Post Box 2713, DK-2100 Copenhagen Ø, Denmark.
Received 15 September 1992; and in revised form 4 January 1993.

*Growth factors*

Porcine TGF$\beta_1$ was purchased from British Biotechnology Ltd, Oxford, England and/or was a gift from Bristol-Meyers-Squibb, Pharamaceutical Research Institute, Seattle, USA. Human recombinant EGF and TGF$\alpha$ was purchased from Bissendorf Biochemicals, Hannover, Germany.

$^{125}$I-labelled TGF$\beta_1$ with a specific activity of 100–180 $\mu$Ci $\mu g^{-1}$ (2.5–4.5 Ci mol$^{-1}$), was purchased from New England Nuclear, Boston, USA. The binding activity of $^{125}$I-labelled TGF$\beta_1$ was checked at regular intervals using the positive control cell line AKR-2B. The $^{125}$I-labelled TGF$\beta_1$ was used within 4 weeks of fresh lot date.

*Radioreceptor assay*

The procedure has been described previously (Massagué & Like, 1985; Massagué, 1987). Cells growing as monolayer culture were plated in 35 mm 6-well dishes, usually at $2-5 \times 10^5$ cells per well, the day before experiments were performed. The cells were washed for 60 min with binding buffer (128 mM NaCl, 5 mM KCl, 5 mM MgSo$_4$, 1.2 mM CaCl$_2$, 50 mM HEPES, pH 7.5 and 2% BSA). After washing, the cells were incubated with 5–10 pM $^{125}$I-labelled TGF$\beta_1$ and increasing levels of native unlabelled TGF$\beta_1$ ranging from 0 to 200 pM, the volume of incubation being adjusted to 1 ml. After 2 h incubation at 20°C the reaction was stopped by washing the plates three times with ice cold binding buffer without albumin. After the final wash, the cells were solubilised in solubilisation buffer (128 mM NaCl, 0.25 mM EDTA, 0.5 mM Tris, pH 7.5 and 1% v/v Triton X-100). An aliquot of the supernatant was counted in a Beckmann II gamma counter (70% efficiency). Protein concentration was determined with the BCA protein kit (Pierce Europe, B.V., Oud Beujerland, The Netherlands) (Smith *et al.*, 1985). Cells growing as floating aggregates or cells easily detectable were assayed, as single cell suspensions, in 1.5 ml sigmacote (Pierce) treated microcentrifuge tubes. Viability after obtaining a single cell suspension, assessed by trypan blue exclusion test, was 90–95%. After incubation the reaction was stopped by centrifuging at 5,500 *g* for 3 min and the cell pellet was resuspended three times in ice cold binding buffer without albumin. After the final wash, the cell pellet was solubilised as above. Maximal binding ($B_{max}$) was calculated as femtomol mg$^{-1}$ protein by Scatchard analysis of the binding data (Scatchard, 1949). Specificity of the binding was determined in specificity experiments with TGF$\beta_1$, EGF and TGF$\alpha$ as the displacing agents. The displacing agents were added at the same time as the $^{125}$I-labelled TGF$\beta_1$.

*Cross-linking*

Washed single cell ($2-5 \times 10^6$) suspensions were incubated with 40 pM $^{125}$I-labelled TGF$\beta_1$ in the presence or absence of a 100-fold excess of unlabelled TGF$\beta_1$. The incubation proceeded for 4 h at 4°C. After the final wash, the cell pellet was resuspended in 950 $\mu$l binding buffer without BSA before 50 $\mu$l of 5 mM cross-linking agent disucinimidyl (DSS) (Pierce, France), freshly dissolved in DMSO, was added. The cross-linking reaction proceeded for 15 min at 4°C and was stopped by centrifuging and washing the pellet in a Tris-containing buffer. Finally the cell pellet was resuspending in 80 $\mu$l solubilisation buffer, 10 $\mu$l cocktail 1 and 10 $\mu$l cocktail 2 as described earlier (Massagué, 1987). The resulting supernatant was boiled for 5 min in sample buffer with 50 mM dithiothreitol (Pierce). One hundred $\mu$g protein/lane was run on a 5, 7 or 10%, 8 × 16 cm SDS-PAGE gel. After staining with Coomassie brilliant blue and destaining, the dried gel was exposed to an X-ray film (Amersham) with an intensifying screen at – 80°C.

*Northern blotting*

RNA was extracted by the single-step acid guanidinium thiocyanate-phenol-chloroform method (Chomczynski & Sacchi, 1987). Ten $\mu$g total RNA samples were electrophoresed through denaturing agarose gels containing 2.2 M formaldehyde, and transferred to nylon membranes (GeneScreen Plus, NEN DuPont) as recommended by the supplier. Radiolabelled probes were prepared by the random priming method (Feinberg & Vogelstein, 1983) using [$\alpha$-$^{32}$P]dCTP and a commerical kit (both from Amersham). The blots were sequentially hybridised with human probes for TGF$\beta$ and for $\beta$-actin. The probes for TGF$\beta_1$ were a 2.0 kb full length cDNA (Kasid *et al.*, 1988) obtained from the American Type Culture Collection (No. 59954) and a 267 bp fragment spanning nucleotides 1773–2040. The probe for TGF$\beta_2$ mRNA was a 442 bp murine fragment of the plasmid pmTGFb2-9a (Miller *et al.*, 1989*b*). The TGF$\beta_3$ probe was a 609 bp murine fragment of the plasmid pmTGF$\beta_3$-IIb (Miller *et al.*, 1989*a*). The TGF$\beta_2$ and TGF$\beta_3$ probes were obtained from Professor H.L. Moses, Vanderbilt University. RNA extracted from murine heart and lung was used as positive controls. The $\beta$-actin probe was a 2.1 kb *Bam*HI fragment of the plasmid pHF$\beta$A-1 (Gunning *et al.*, 1983). The membranes were prehybridised, hybridised and washed as recommended by the supplier, and exposed to an X-ray film at – 80C with an intensifying screen.

### Results

*Receptor binding studies*

Saturation of the receptors were reached with a TGF$\beta_1$ concentration in the range of 50 to 100 pM (exemplified in Figure 1). Non-specific binding, defined as the cell associated radioactivity in the presence of a large excess of unlabelled TGF$\beta_1$, was relatively high but was an inverse function of binding capacity – 23% in AKR-2B with a $B_{max}$ of 70 femtomol mg$^{-1}$ protein and 70% in GLC 19, assayed as a single cell suspension, with a $B_{max}$ of 4.5 femtomol mg$^{-1}$ protein. This relationship has also been described in other cell types (Massagué, 1987). As about half the cell lines grew as floating aggregates and half as monolayer cultures, we chose to relate all binding data to protein concentration. Scatchard analysis of the binding data showed that cells bound between 5 and 27 fmol mg$^{-1}$ protein with a $K_D$ of 16–40 pM (Table I).

The specificity of the TGF$\beta$-r/ligand binding was determined using different displacing agents. The specificity of $^{125}$I-labelled TGF$\beta_1$ binding to GLC 3 is shown in Figure 2. It was found that EGF and TGF$\alpha$, which both binds to the EGF-receptor (Carpenter *et al.*, 1983) did not displace $^{125}$I-labelled TGF$\beta_1$. For all cells tested and found positive in the radioreceptor assay, binding was in all cases specific and saturable.

*Cross-linking studies*

TGF$\beta_1$ binding to the receptors was further visualised by cross-linking the ligand-receptor complex with DSS. Figure 3 illustrates the affinity labelling results of eight SCLC-cell lines. Following electrophoresis on a SDS-PAGE gel, specific TGF$\beta_1$ binding was seen as bands with calculated $M_r$ of 65,000, 90,000 and 280,000; these bands correspond to the type I, II and III TGF$\beta$-r. The presence of excess unlabelled TGF$\beta_1$ resulted in the disappearance of these bands, demonstrating that the binding was specific. Seven SCLC-cell lines were TGF$\beta$-r positive in the affinity labelling experiments (Table I).

*Northern blotting studies*

The cell lines were examined for the production of TGF$\beta$ mRNA. Figure 4a illustrates a Nothern blot analysis of 20 cell lines using the full length TGF$\beta_1$ cDNA probe. The TGF$\beta_1$ mRNa is seen as a band of approximately 2.5 kb. Results using the 267 bp TGF$\beta_1$ fragment was similar (data not shown). Nineteen cell lines were hybridised with probes

**Figure 1** TGFβ binding to a SCLC cell line. CPH 54A was incubated with 5 pM $^{125}$I-labelled TGF$\beta_1$ and increasing concentrations of unlabelled TGF$\beta_1$ as described in Materials and methods. Insert: Scatchard plot of the receptor specific binding per 100 µg protein (B) and the free ligand concentration (F). $K_D$ is given by the slope of the curve. $B_{max}$ is given by the x-axis intercept. ■ Total binding; ○ Receptor specific binding; ▲ Non specific binding.

**Figure 2** Specificity of TGF$\beta_1$ binding. GLC 3 was incubated with 5 pM $^{125}$I-labelled TGF$\beta_1$ and displaced with increasing levels of: ■ EGF; ○ TGFα; ▼ TGF$\beta_1$: Binding is expressed in % of maximal specific binding, 100% being 7.300 CPM mg$^{-1}$ protein.

for TGF$\beta_2$ and TGF$\beta_3$. In two cell lines (Figure 4b) TGF$\beta_2$ mRNA was detected as a faint band of 3.9 kb. TGF$\beta_2$ mRNa, size approximately 3.5 kb, was found in seven cell lines (Figure 4c). Blots were rehybridised with the β-actin probe to demonstrate equal loading in all lanes. The intensity of staining with the TGFβ probe therefore semiquantifies the TGFβ mRNA content.

The results for all binding data, affinity labelling and Northern blot analysis are summarised in Table I. Six of the TGFβ-r positive cell lines also expressed TGFβ (Table I).

### Discussion

This is to our knowledge the first study in which a broad panel of SCLC cell lines has been studied for the presence of TGFβ-receptors and the expression of TGFβ mRNA. The panel of SCLC cell lines, established in five different laboratories and cultured in different media, is probably a representative cross-section of SCLC cell lines. In order to standardise our radioreceptor assay, we carried out the normal time- and temperature course experiments as described

**Table I** Summary of the TGFβ receptor and TGFβ expression in SCLC cell lines

| Cell line | Bound TGFβ fmol mg$^{-1}$ | $K_D$ (pM) | Receptor I | Receptor II | Receptor III | TGFβ mRNA 1 | TGFβ mRNA 2 | TGFβ mRNA 3 |
|---|---|---|---|---|---|---|---|---|
| DMS 53 | – | – | – | – | – | + | (+) | – |
| DMS 79 | – | – | – | – | – | + | – | – |
| DMS 92 | – | – | – | – | – | – | – | – |
| DMS 114 | 26.6 ± 2.1[a] | 40.3 ± 4.5 | – | – | + | + | (+) | + |
| DMS 153 | – | – | – | – | – | + | – | – |
| DMS 273 | 5.2 ± 0.5 | 20.6 ± 2.3 | – | (+) | + | + | – | (+) |
| DMS 406 | – | – | – | – | – | – | – | – |
| DMS 456 | – | – | – | – | – | – | – | – |
| GLC 2 | – | – | – | – | – | – | – | + |
| GLC 3 | 10.7 ± 1.1 | 22.7 ± 1.4 | – | – | + | + | – | + |
| GLC 14 | – | – | – | – | – | – | – | – |
| GLC 16 | – | – | + | + | – | – | – | – |
| GLC 19 | 4.5 ± 1.1 | 16.1 ± 4.4 | + | + | – | + | – | – |
| GLC 26 | – | – | – | – | – | – | – | – |
| GLC 28 | – | – | – | – | – | – | – | – |
| 24H | – | – | – | – | – | – | – | + |
| 86M1 | – | – | – | – | – | NT | NT | NT |
| NCI H69 | – | – | – | – | – | – | – | – |
| NCI N417 | – | – | – | – | – | + | NT | NT |
| CPH 54A | 27.6 ± 2.6 | 25.2 ± 6.2 | + | + | + | + | – | (+) |
| CPH 54B | 21.3 ± 3.9 | 23.0 ± 6.2 | + | + | + | + | – | + |

$B_{max}$ and $K_D$ were determined as described in the text. TGFβ-r was detected by cross-linking studies. TGF$\beta_1$, TGF$\beta_2$ and TGF$\beta_3$ mRNA was detected by Northern blotting. –: Negative. +: TGFβ receptor or mRNA detected. (+): Faint band. a: Mean of 3–4 values ± s.d. NT: not tested.

**Figure 3** Affinity labelling of the TGF$\beta$ receptor. Cells were incubated with $^{125}$I-labelled TGF$\beta_1$ as described in Materials and methods, the receptor complex was cross-linked with DSS and size fractionated on either **a**, 5% SDS-PAGE gel to demonstrate the high molecular weight betaglycan (type III receptor) or **b**, 10% gel to demonstrate the receptor type I and II. **c**, 7% gel for 3 GLC cell lines. For each cell line two lines were run: – 40 pM $^{125}$I-labelled TGF$\beta_1$; + 100-fold excess of unlabelled TGF$\beta_1$. Molecular weight standards from Bio-Rad were co-electroforesed. Roman numerals indicates the type I and II TGF$\beta$-r. Arrow indicate the TGF$\beta$ type III receptor.

elsewhere (Frolik *et al.*, 1984; Tucker *et al.*, 1984), and found that the binding was stable for a prolonged period of time at 20°C. Furthermore we found that the receptors could not be demonstrated if the protein concentration was lower than 150 $\mu$l ml$^{-1}$. We have previously demonstrated, in the same cell lines, studying the EGF-receptor that this critical protein level was also required to detect the EGF-receptor (Damstrup *et al.*, 1992). Studies on other receptor systems such as the estrogen receptor has also demonstrated this critical protein limit (Skovgaard Poulsen, 1981). Therefore, to avoid underestimating binding capacity or falsely classify a SCLC cell line as TGF$\beta$-r negative, we only drew conclusions on the TGF$\beta$-receptor state in a cell line if the protein concentration was in the range of 200–600 $\mu$g ml$^{-1}$.

Analysis of the binding data demonstrated that Scatchard plots in some cell lines were curved near saturation of the receptors. However, it was not possible, with the ligand program developed by Munson and Rodbard (Munson & Rodbard, 1980), to resolve the Scatchard plots into two or more compartments. Other investigators have, in normal rat

**Figure 4** Northern blot analysis of TGFβ expression in SCLC. **a**, Probed with the TGF$\beta_1$ cDNA, **b**, with TGF$\beta_2$ and **c**, with TGF$\beta_3$. The resulting 7 day autoradiography is shown. The 2.5 kb TGF$\beta_1$ mRNA, the 3.9 kb TGF$\beta_2$ mRNA and the 3.5 kb TGF$\beta_3$ mRNAs are indicated. Transcript size for TGF$\beta_1$ was determined with reference to the 18 and 28S bands. Transcript sizes for TGF$\beta_2$ and TGF$\beta_3$ were determined with reference to mRNA molecular weight markers with band sizes 1.4, 2.4, 4.4, 7.5 and 9.5 kb (Life Technologies). The β-actin probing indicate that the lanes were loaded equally. The band seen in all cell lines probed with TGF$\beta_1$ represents non-specific binding the 28S (4.8 kb) ribosomal band. *Murine lung mRNA is included as a positive control for the TGF$\beta_2$ and TGF$\beta_3$ probes.

kidney cell (NRK), also only demonstrated one class of TGFβ receptors, despite the fact that cross-linking studies with NRK cells have demonstrated that these cells express type I, II and III receptors (Wakefield, 1987; Massagué & Like, 1985; Segarini *et al.*, 1987). Resolving the data with a single class receptor from the linear part of the Scatchard plot demonstrated high affinity receptors in six SCLC cell lines (Figure 1, Table I). The dissociation constant was in all cases characteristic for TGFβ binding (Massagué, 1987; Wakefield, 1987). Maximal binding varied from 4.5 to 27.5 fmol $mg^{-1}$ protein. Binding of $^{125}$I-labelled TGF$\beta_1$ to the positive cells was specific as only TGFβ could displace the labelled TGF$\beta_1$. EGF and TGFα did not influence TGFβ binding (Figure 2).

The results obtained from the radioreceptor assay and the displacement studies demonstrated that a large proportion of the SCLC cell lines examined carried specific high affinity TGFβ-r. Our results are in part corroborated as one of these cell lines, GLC 19, has previously been reported to be growth inhibited by TGFβ (Lagadec *et al.*, 1991). However, in the cited study the cells were not examined for the presence of TGFβ-r.

To verify that the binding of TGF$\beta_1$ was in fact to the TGFβ-r, the cell lines were tested by cross-linking. After size fractionation on SDS-PAGE gels, all cell lines found to be TGFβ-r positive in the radioreceptor assay also displayed one or more specific bands with calculated $M_r$ = 65,000, 90,000 and 280,000 (Figure 3). These sizes include reduced TGFβ with a $M_r$ of approximately 12,000. The $M_r$ of the corresponding receptors is therefore 53,000, 78,000 and 270,000. The TGFβ receptors have previously been reported as having these calcualted molecular weight (Massagué, 1987; Massagué, 1990). This provides further evidence that TGF$\beta_1$ binding was to the TGFβ-r. One cell line, GLC 16, expressed the type I and II TGFβ-r in this assay. We could, however not demonstrate the receptor in the radioreceptor assay, even using a very high protein concentration (> 800 μg $ml^{-1}$). The binding capacity in this cell line could be so low that it was below the detection limit in the radioreceptor assay. In the same cell line, we have found that the receptor was functional in that exogenously added TGF$\beta_1$ acted as a growth inhibitor (Nørgård, P., unpubished observation).

We also examined the expression of TGFβ mRNAs in the panel. In 10/20 SCLC cell lines TGF$\beta_1$ mRNA could be detected (Table I, Figure 4a). In GLC 3 and faintly in DMS 153 an additional band of 1.7 kb was found, the nature of this band is unclear. A mRNA with this approximate size has also been found in male mice germ cells (Watrin *et al.*, 1991).

We examined 19 of the SCLC cell lines with a probe for TGF$\beta_2$ mRNA (Figure 4b), and in two cell lines (DMS 53 and DMS 114) a single transcript of 3.9 kb was demonstrated. This is in accordance with one of the TGF$\beta_2$ transcripts reported in other human cell lines (Mori *et al.*, 1990), whereas none of the additional TGF$\beta_2$, mRNAs reported (Jakowlew *et al.*, 1991; Mori *et al.*, 1990; Miller *et al.*, 1989*b*) were detected in the investigated cell lines.

We have previously examined 15 of the cell lines in our panel for expression of phosphorylated retinoblastoma gene product (pRb) (Rygaard *et al.*, 1990). Only the two cell lines (DMS 53 and DMS 114) in which TGF$\beta_2$ mRNA was detected, were found to express pRb, and immunocytochemistry demonstrated nuclear localisation of pRb (Rygaard, K., unpublished observation). Other studies (Templeton *et al.*, 1991) have suggested that pRb is functional only when phosporylated and located in the nucleus. It has recently been reported that the pRb activates the expression of TGF$\beta_2$ (Kim *et al.*, 1992). Provided that the characteristics of pRb in DMS 53 and DMS 114 indicates that the protein is functional, our finding that TGF$\beta_2$ was detected exclusively in the two cell lines also expressing a 'functional' pRb, agrees with the concept that expression of TGF$\beta_2$ is activated by pRb.

Seven cell lines expressed the 3.5 kb TGF$\beta_3$ mRNA (Figure 4c), corresponding to the reported size in other human malignant tissue (Dijke *et al.*, 1988). Two of these cell lines (GLC 2 and 24H) also expressed a transcript with a size of approximately 2.5 kb, which is the transcript size of TGF$\beta_1$ mRNA, however, these two cell lines did not express TGF$\beta_1$ mRNA.

The probing for TGF$\beta_2$ and TGF$\beta_3$ was performed with a murine probe, and there may not be perfect homology to the human mRNA. This implies that additional cell lines could be positive following probing with a human probe. Taken together a total of 12 of the 20 examined SCLC cell lines expressed TGF$\beta$ mRNA. This finding is in contrast to earlier studies, where a few cell lines have been examined and found to be TGF$\beta$ mRNA negative (Söderdahl *et al.*, 1988; Derynck *et al.*, 1987; Lagadec *et al.*, 1991). In one of these studies (Lagadec *et al.*, 1991) the examined cell lines included GLC 14, 16 and 19, NCI H69 and N417, all these were TGF$\beta$ mRNA negative. These cell lines were also included in our panel, but we found expression of TGF$\beta_1$ mRNA in GLC 19 and NCI N417. This transcript was detected as a 2.5 kb band using both the full length cDNA and the 267 bp TGF$\beta_1$ fragment. The difference betwen our results and the previous reported study (Lagadec *et al.*, 1991), is not apparent, but could be due to a difference in sensitivity.

Our results based on a panel of 21 SCLC cell lines have demonstrated that TGF$\beta$ receptors were present in seven of 21 SCLC cell lines and more than half of the cells examined expressed TGF$\beta$ mRNA. About half of the examined cell lines grew as monolayer cultures and half as floating aggregates, but we could not detect any statistical difference between the growth morphology and the expression of TGF$\beta$-r or TGF$\beta$ mRNAs (Chi-square test with Yates correction and Fisher's exact test, $P < 0.2$). Coexpression of TGF$\beta$-r and the ligand was found in six cell lines. These cell lines therefore have the possibility of an autocrine growth regulation. The question whether the SCLC cell lines produce TGF$\beta$ protein and if this is biologically active is currently being investigated.

This work was supported by the Danish Cancer Society, the Danish Research Academy, the Haench's foundation, the Henriksen's foundation, the Madsen foundation and the Vissing's foundation. The authors thank Mrs C. Jespersen and J. Rørhman for technical assistance.

### References

ANDRES, J.L., STANLEY, K., CHEIFETZ, S. & MASSAGUÉ, J. (1989). Membrane-anchored and soluble forms of betaglycan, a polymorphic proteoglycan that binds transforming growth factor-$\beta$. *J. Cell Biol.*, **109**, 3137–3145.

BERNARD, J.A., LYONS, R.M. & MOSAES, H.L. (1990). The cell biology of transforming growth factor $\beta$. *Biochim. Biophys. Acta*, **1032**, 79–87.

BEPLER, G., JAQUES, G., NEUMANN, K., AUMÜLLER, G., GROPP, C. & HAVEMANN, K. (1987). Establishment, growth properties, and morphological characteristics of permanent human small cell lung cancer cell lines. *J. Cancer Res. Clin. Oncol.*, **113**, 31–40.

BERENDSEN, H.H., DE LEIJ, L., DE VRIES, E.G.E., MESANDER, G., MULDER, N.H., DE JONG, B., BUYS, C.H.C.M., POSTMUS, P.E., POPPEMA, S., SLUITER, H.J. & THE, H.T. (1988). Characterization of three small cell lung cancer cell lines established from one patient during longitudinal follow-up. *Cancer Res.*, **48**, 6891–6899.

BERGH, J. (1988). The expression of the platelet-derived and transforming growth factor genes in human non-small lung cancer cell lines is related to tumor stroma formation in nude mice tumors. *Am. J. Pathol.*, **133**, 434–439.

BOYD, F.T. & MASSAGUÉ, J. (1989). Transforming growth factor-$\beta$ inhibition of epithelial cell proliferation linked to the expression of a 52-kDa membrane receptor. *J. Biol. Chem.*, **264**, 2272–2278.

CARNEY, D.N., GAZDAR, A.F., BEPLER, G., GUCCION, J.G., MARANGOS, P.J., MOODY, T.W., ZWEIG, M.H. & MINNA, J.D. (1985). Establishment and identification of small cell lung cancer cell lines having classic and variant features. *Cancer Res.*, **45**, 2913–2923.

CARPENTER, G., STOSCHECK, C.M., PRESTON, Y.A. & DE LARCO, J.E. (1983). Antibodies to the epidermal growth factor receptor blocks the biological activities of sarcoma growth factor. *Proc. Natl Acad. Sci. USA*, **80**, 5627–5630.

CHEIFETZ, S., LING, N., GUILLEMIN, R. & MASSAGUÉ, J. (1988). A surface component on $GH_3$ pituitary cells that recognizes transforming growth factor-$\beta$, activin, and inhibin. *J. Biol. Chem.*, **263**, 17225–17228.

CHOMCZYNSKI, P. & SACCHI, N. (1987). Single-step method of RNA isolation by acid guanidinium thiocyanate-phenol-chloroform extraction. *Anal. Biochem.*, **162**, 156–159.

DAMSTRUP, L., RYGAARD, K., SPANG-THOMSEN, M. & SKOVGAARD POULSEN, H. (1992). Expression of the epidermal growth factor receptor in human small cell lung cancer cell lines. *Cancer Res.*, **52**, 3089–3093.

DE LEIJ, L., POSTMUS, P.E., BUYS, C.H.C.M., ELEMA, J.D., RAMAEKERS, F., POPPEMA, S., BROUWER, M., VAN DER VEEN, A.Y., MESANDER, G. & THE, T.H. (1985). Characterization of three new variant type cell lines derived from small cell carcinoma of the lung. *Cancer Res.*, **45**, 6024–6033.

DE MARTIN, R., HAENDLER, B., HOFER-WARBINEK, R., GAUGITSCH, H., WRANN, M., SCHLUSENER, H., SEILERT, J.M., BODMER, S., FONTANA, A. & HOFER, E. (1987). Complementary DNA for human glioblastoma-derived T cell suppressor factor, a novel member of the transforming growth factor-$\beta$ gene family. *EMBO J*, **6**, 3673–3677.

DERYNCK, R., JARRETT, J.A., CHEN, E.Y., EATON, D.H., BELL, J.R., ASSOCIAN, R.K., ROBERTS, A.B., SPORN, M.B. & GOEDDEL, D.V. (1985). Human transforming growth factor-$\beta$ complementary DNA sequence and expression in normal and transformed cells. *Nature*, **316**, 701–705.

DERYNCK, R., GOEDDEL, D.V., ULLRICH, A., GUTTERMAN, J.U., WILLIAMS, R.D., BRINGMAN, T.S. & BERGER, W.H. (1987). Synthesis of messenger RNAs for transforming growth factor $\alpha$ and $\beta$ and the epidermal growth factor receptor by human tumors. *Cancer Res.*, **47**, 707–712.

DIJKE, P.T., HANSEN, P., IWATA, K.K., PIELER, C. & FOULKES, J.G. (1988). Identification of another member of the transforming growth factor type $\beta$ gene family. *Proc. Natl Acad. Sci. USA*, **85**, 4715–4719.

ENGELHOLM, S.AA., SPANG-THOMSEN, M., VINDELØV, L.L., BRÜNNER, N., NIELSEN, M.H., HIRSCH, F., NIELSEN, A. & HANSEN, H.H. (1986). Comparison of characteristics of human small cell carcinoma of the lung in patients, *in vitro* and transplanted into nude mice. *Acta Path. Microbiol. Immunol. Scand.*, **94**, 325–336.

FEINBERG, A.P. & VOGELSTEIN, B. (1983). A technique for radiolabelling DNA restriction endonuclease fragments to high specific activity. *Anal. Biochem.*, **132**, 6–13.

FROLIK, C.A., WAKEFIELD, L.M., SMITH, D.M. & SPORN, M.B. (19840. Characterization of a membrane receptor for transforming growth factor-$\beta$ in normal rat kidney fibroblasts. *J. Biol. Chem.*, **259**, 10995–11000.

GUNNING, P., PONTE, P., OKAYAMA, H., ENGEL, J., BLAU, H. & KEDES, L. (1983). Isolation and characterization of full-length cDNA clones for human $\alpha$-, $\beta$- and (gamma)-actin mRNAs: skeletal but not cytoplasmic actins have an amino-terminal cysteine that is subsequently removed. *Mol. Cell Biol.*, **3**, 787–795.

JAKOWLEW, S.B., DILLARD, P.J., SPORN, M.B. & ROBERTS, A.B. (1988). Complementary deoxyribonucleic acid cloning of an mRNA encoding transforming growth factor-beta 4 from chicken embryo chondrocytes. *Mol. Endocrinol.*, **2**, 1186–1195.

JAKOWLEW, S.J., DILLARD, P.J., WINOKUR, T.S., FLANDERS, K.C., SPORN, M.B. & ROBERTS, A.B. (1991). Expression of transforming growth factor-βs 1-4 in chicken embryo chondrocytes and myocytes. *Develop. Biol.*, **143**, 135–148.

KASID, A., BELL, G.I. & DIRECTOR, E.P. (1988). Effects of transforming growth factor-β on human lymphokine-activated killer cell precursors. Autocrine inhibition of cellular proliferation and differentiation to immune killer cells. *J. Immunol.*, **141**, 690–698.

KIM, S.J., WAGNER, S., LIU, F., OREILLY, M.A., ROBBINS, P.D. & GREEN, M.R. (1992). Retinoblastoma gene product activates expression of the human TGF-β2 gene through transcription factor ATF-2. *Nature*, **358**, 331–334.

LAGADEC, P.F., SARAYA, K.A. & BALKWILL, F.R. (1991). Human small-cell lung-cancer cells are cytokine-resistant but NK/LAK-sensitive. *Int. J. Cancer*, **48**, 311–317.

LAIHO, M., DECAPRIO, J.A., LUDLOW, J.W., LIVINGSTON, D.M. & MASSAGUÉ, J. (1990). Growth inhibition by TGF-(beta) linked to suppression of retinoblastoma protein phosphorylation. *Cell*, **62**, 175–185.

LIN, H.Y., WANG, X.-F., NG-EATON, E., WEINBERG, R.A. & LODISH, H.F. (1992). Expression cloning of the TGF-β type II receptor, a functional tranmsmembrane serine/threonine kinase. *Cell*, **68**, 775–785.

MACKAY, K. & DANIELPOUR, D. (1991). Novel 150- and 180-kDa glycoproteins that bind transforming growth factor (TGF)-β1 but not TGF-β2 are present in several cell lines. *J. Biol. Chem.*, **266**, 9907–9911.

MADISEN, L., WEBB, N.R., ROSE, T.M., MARQUARDT, H., IKEDA, T., TWARDZIK, D.R., SEYEDIN, S.M. & PURCHIO, A.F. (1988). Transforming growth factor-β2: cDNA cloning and sequence analysis. *DNA*, **7**, 1–8.

MASSAGUÉ, J. Identification of receptors for type-β transforming growth factor. In: *Methods in Enzymology*, Colowick, S.P. & Kaplan, N.O. (eds). Academic Press, 1987, p. 174–195.

MASSAGUÉ, J. (1990). The transforming growth factor-β family. *Annu. Rev. Cell Biol.*, **6**, 597–641.

MASSAGUÉ, J. & LIKE, B. (1985). Cellular receptors for type β transforming growth factor. *J. Biol. Chem.*, **260**, 2636–2645.

MILLER, D.A., LEE, A., MATSUI, Y., CHEN, E.Y., MOSES, H.L. & DERYNCK, R. (1989*a*). Complementary DNA cloning of the murine transforming growth factor-β3 (TGFβ3) precursor and the comparative expression of TGFβ3 and TGFβ1 messenger RNA in murine embryos and adult tissues. *Mol. Endocrinol.*, **3**, 1926–1934.

MILLER, D.A., LEE, A., PELTON, R.W., CHEN, E.Y., MOSES, H.L. & DERYNCK, R. (1989*b*). Murine transforming growth factor-β2 cDNA sequence and expression in adult tissues and embryos. *Mol. Endocrinol.*, **3**, 1108–1114.

MORI, H., MAKII, M., OISHI, K., JAYE, M., IGARASHI, K., YOSHIDA, O. & HATANAKA, M. (1990). Increased expression for basic fibroblast growth factor and transforming growth factor beta type β2 in human benign prostatic hyperplasia. *Prostate*, **16**, 71–80.

MUNSON, P.J. & RODBARD, D. (1980). Ligand: a versatile computerized approach for characterization of ligand-binding systems. *Analytical Biochem.*, **107**, 220–239.

O'GRADY, P., KUO, M.-D., BALDASSARE, J.J., HUANG, S.S. & HUANG, J.S. (1991). Purification of a new type high molecular weight receptor (type V receptor) of transforming growth factor β (TGFβ) from bovine liver. *J. Biol. Chem.*, **266**, 8583–8589.

PELTON, R.W., SAXENA, B., JONES, M., MOSES, H.L. & GOLD, L.I. (1991). Immunohistochemical localization of TGFβ1, TGFβ2, and TGFβ3 in the mouse embryo: expression patterns suggest multiple roles during embryonic development. *J. Cell Biol.*, **115**, 1091–1105.

PELTON, R.W., MOSES, H.L. (1990). The beta-type transforming growth factor. Mediators of cell regulation in the lung. *Am. Rev. Respir. Dis.*, **142**, S31–S35.

PETTENGILL, O.S., SORENSON,M G.D., WURSTER-HILL, D., CURPHEY, T.J., NOLL, W.W., CATE, C.C. & MAURER, L.H. (1980). Isolation and growth characteristics of continuous cell lines from small-cell carcinoma of the lung. *Cancer*, **45**, 906–918.

RYGAARD, K., SORENSON, G.D., PETTENGILL, O.S., CATE, C.C. & SPANG-THOMSEN, M. (1990). Abnormalities in structure and expression of the retinoblastoma gene in small cell lung cancer cell lines and xenografts in nude mice. *Cancer Res.*, **50**, 5312–5317.

SCATCHARD, G. (1949). The attractions of proteins for small molecules and ions. *Annals NY Acad. Science*, **51**, 660–672.

SEGARINI, P.R., ROBERTS, A.B., ROSEN, D.M. & SEYEDIN, S.M. (1987). Membrane binding characteristics of two forms of transforming growth factor-β. *J. Biol. Chem.*, **262**, 14655–14662.

SKOVGAARD POULSEN, H. (1981). Oestrogen receptor assay – limitations of the method. *Eur. J. Cancer*, **17**, 495–501.

SMITH, P.K., KROHN, R.L. & HERMANSON, G.T. (1985). Measurement of protein using bicinichoninic acid. *Analytical Biochem.*, **150**, 76–85.

SÖDERDAHL, G., BETSCHOLTZ, C., JOHANSSON, A., NILSSON, K. & BERGH, J. (1988). Differential expression of platelet-derived growth factor and transforming growth factor genes in small- and non-small-cell human lung carcinoma lines. *Int. J. Cancer*, **41**, 636–641.

TEMPLETON, D.J., PARK, S.H., LANIER, L. & WEINBERG, R.A. (1991). Nonfunctional mutants of the retinoblastoma protein are characterized by defects in phosphorylation, viral oncoprotein association, and nuclear tethering. *Proc. Natl Acad. Sci. USA*, **88**, 3033–3037.

TUCKER, R.F., BRANUM, E.L., SHIPLEY, G.D., RYAN, R.J. & MOSES, H.L. (1984). Specific binding to cultured cells of $^{125}$I-labelled type β transforming growth factor from human platelets. *Proc. Natl Acad. Sci. USA*, **81**, 6757–6761.

WAKEFIELD, L.M. An assay for type-β transforming growth factor receptor. In: *Methods in Enzymology*, Colowick, S.P. & Kaplan, N.O. (eds). Academic Press, 1987, p. 167–173.

WANG, X.-F., LKIN, H.Y., NG-EATON, E., DOWNWARD, J., LODISH, H.F. & WEINBERG, R.A. (1992). Expression cloning and characterization of the TGF-β type III receptor. *Cell*, **67**, 797–805.

WATRIN, F., SCOTTO, L., ASSOIAN, R.K. & WOLGEMUTH, D.J. (1991). Cell lineage specificity of expression of the murine transforming growth factor $\beta_3$ and transforming growth factor $\beta_1$ genes. *Cell Growth Differ.*, **2**, 77–83.